# Quantification of Moss-Associated Cyanobacteria Using Phycocyanin Pigment Extraction

**DOI:** 10.3389/fmicb.2020.611792

**Published:** 2021-01-05

**Authors:** Marie Renaudin, Romain Darnajoux, Jean-Philippe Bellenger

**Affiliations:** ^1^Centre Sève, Département de Chimie, Université de Sherbrooke, Sherbrooke, QC, Canada; ^2^Department of Geosciences, Princeton University, Princeton, NJ, United States

**Keywords:** phycocyanin, cyanobacteria, nitrogen fixation, feather moss, boreal forest

## Abstract

In the boreal forest, cyanobacteria can establish associations with feather moss and realize the biological nitrogen fixation (BNF) reaction, consisting in the reduction of atmospheric dinitrogen into bioavailable ammonium. In this ecosystem, moss-associated cyanobacteria are the main contributors to BNF by contributing up to 50% of new N input. Current environmental changes driven by anthropogenic activities will likely affect cyanobacteria activity (i.e., BNF) and populations inhabiting mosses, leading to potential important consequences for the boreal forest. Several methods are available to efficiently measure BNF activity, but quantifying cyanobacteria biomass associated with moss is challenging because of the difficulty to separate bacteria colonies from the host plant. Attempts to separate cyanobacteria by shaking or sonicating in water were shown to be poorly efficient and repeatable. The techniques commonly used, microscopic counting and quantitative PCR (qPCR) are laborious and time-consuming. In aquatic and marine ecosystems, phycocyanin (PC), a photosynthesis pigment produced by cyanobacteria, is commonly used to monitor cyanobacteria biomass. In this study, we tested if PC extraction and quantification can be used to estimate cyanobacteria quantity inhabiting moss. We report that phycocyanin can be easily extracted from moss by freeze/thaw disturbance of cyanobacteria cells and can be quickly and efficiently measured by spectrofluorometry. We also report that phycocyanin extraction is efficient (high recovery), repeatable (relative SD < 13%) and that no significant matrix effects were observed. As for aquatic systems, the main limitation of cyanobacteria quantification using phycocyanin is the difference of cellular phycocyanin content between cyanobacteria strains, suggesting that quantification can be impacted by cyanobacteria community composition. Nonetheless, we conclude that phycocyanin extraction and quantification is an easy, rapid, and efficient tool to estimate moss-associated cyanobacteria number.

## Introduction

Mosses are cryptogamic plants found in a very large range of terrestrial and aquatic ecosystems around the globe (Fogg, 1998). Mosses are particularly abundant in the boreal forest, the largest terrestrial biome on Earth (DeLuca and Boisvenue, 2012), where they can cover up to 70–100% of the ground (Oechel and Van Cleve, 1986). Mosses affect microbial activity in soil by regulating soil temperature and moisture (Luthin and Guymon, 1974; Gornall et al., 2007) and by releasing nutrients, such as dissolved organic carbon and potassium (Wilson and Coxson, 1999). In the boreal forest, feather mosses also play an important role in the carbon (C) and nitrogen (N) cycles, and contribute up to a third of the total forest primary productivity (DeLuca et al., 2002; Turetsky, 2003; Turetsky et al., 2010; Wardle et al., 2011; Liu et al., 2020). In addition, the reaction of biological nitrogen fixation (BNF), catalyzed by diazotrophic bacteria associated with feather moss, can contribute up to 50% of new N inputs (Turetsky et al., 2012; Rousk and Michelsen, 2017) on par with atmospheric deposition. Several cyanobacteria genera (e.g., *Calothrix*, *Cylindrospermum*, *Fischerella*, *Nostoc*, and *Stigonema*) were found living epiphytically on boreal feather mosses (DeLuca et al., 2002; Gentili et al., 2005; Houle et al., 2006; Zackrisson et al., 2009; Ininbergs et al., 2011) and are considered the main contributors to moss BNF in the boreal forest (Leppänen et al., 2013).

Several studies reported a positive linear relationship between cyanobacteria abundance and BNF activity in boreal mosses and suggest that moss can regulate cyanobacteria colonization according to their N needs (DeLuca et al., 2007; Gundale et al., 2011; Rousk et al., 2013, 2017). Beside moss N demand, other environmental parameters can affect moss-cyanobacteria associations (cyanobacteria abundance and BNF), such as moisture, temperature, heavy metal, and phosphorus deposition (Gundale et al., 2012; Rousk et al., 2017; Jean et al., 2018; Scott et al., 2018). With global warming and the development of human activities at northern latitudes, boreal forests, and feather mosses will undergo important changes in climatic conditions (i.e., average temperature and water regime) and atmospheric deposition (nutrients). Indeed, it has been predicted that the average annual temperature in the boreal forest will increase by 2°C by 2050 (Price et al., 2013). This will cause the extension of the growth season length (Ouranos, 2015) and, combined with CO_2_ increase, will impact boreal forest primary productivity, C cycle, and N demand (Lloyd and Bunn, 2007; Sigurdsson et al., 2013; Tagesson et al., 2020). Evaluating how these environmental changes will affect moss-associated nitrogen fixing bacteria is essential to help better predict the response of the boreal forest to global change. Thus, consistent and rigorous methods to characterize how the moss-associated cyanobacteria biomass and BNF are affected by environmental factors are needed.

Cyanobacterial BNF activity in moss can be easily assessed, indirectly, using the reaction of acetylene reduction into ethylene as a proxy (i.e., Acetylene Reduction Assay (ARA) method, Hardy et al., 1968) or directly, by the incorporation of ^15^N tracer (Leppänen et al., 2013; Jean et al., 2018). Accurately quantifying cyanobacteria quantity, on the other hand, remains challenging. Three approaches have been used to estimate cyanobacteria quantity associated with moss. In the first approach, cyanobacteria are directly counted on whole moss shoots or leaves under an epifluorescence microscope (DeLuca et al., 2007; Gundale et al., 2011; Rousk et al., 2013). This approach is laborious, time consuming and, because cyanobacteria are often grouped into multilayer colonies located within leaf incurves (DeLuca et al., 2002), accurately counting individual cells is complicated. Moreover, counting is only performed on a relatively small number of moss shoots and leaves, which makes it difficult to extrapolate to a cyanobacteria quantity per surface *in situ*. In the second approach, cyanobacteria colonies are extracted from moss prior to being counted under a fluorescence microscope, as in the first approach. This technique allows estimating cyanobacteria number on a larger amount of moss stems with reducing errors due to variation in cyanobacteria density between stems. Different cyanobacteria separation techniques have been explored. Sonication was used to isolate cyanobacteria from the moss (Lindo and Whiteley, 2011) but it has been reported to lead to bacteria cell lysis (Reksten, 2014), whereas shaking or vortexing moss shoots immersed in distilled water (Jean et al., 2012; Rousk et al., 2017) result in variable extraction efficiencies that can only be overcome by performing a very large number of replicates. Moreover, as for the first approach, counting colonies after extraction only provides rough estimates of cyanobacteria quantity and is probably biased by differences of extraction efficiencies between cyanobacteria genera (Whiteley and Gonzalez, 2016). The last approach, more rarely used, relies on molecular biology techniques, such as quantitative PCR (qPCR), to estimate global cyanobacteria quantity or genera/species relative abundance (Warshan et al., 2016). Primers targeting the cyanobacterial 16S rRNA gene CYA 359F and CYA 781Ra/Rb (Nübel et al., 1997) are usually used. The qPCR approach is relatively sensitive but time consuming, costly and is based on primers selectivity, which can bias cyanobacteria quantification in moss samples. Moreover, variation in 16S rRNA gene copy number has been demonstrated for several cyanobacteria genera (Engene et al., 2010; Engene and Gerwick, 2011) and could affect qPCR results when studying mixed-genera cyanobacteria communities present in moss. More recently, Arróniz-Crespo et al. (2014) proposed an alternative for the quantification of cyanobacteria number living on moss based on the extraction and quantification of the echinenone pigment by HPLC separation coupled with a photodiode array detector.

**Figure 1:**
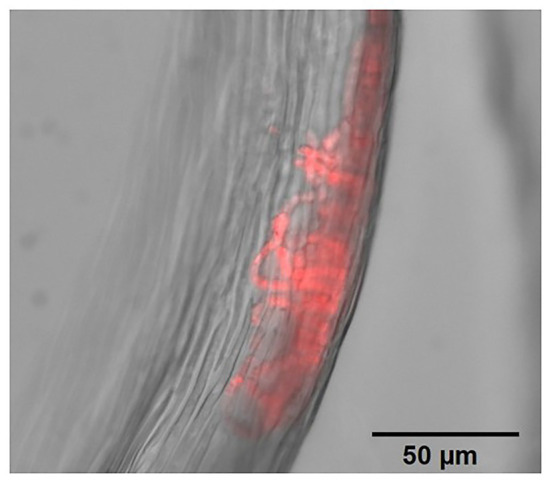
Filamentous cyanobacteria colonies (phycocyanin fluorescence in red) within *Ptilium crista-castrensis* leaves, observed under an epifluorescence microscope (Zeiss Axio Observer Z1 equipped with a Zeiss Axiocam 506 mono, objective 40X/0.95NA). For widefield fluorescence, an excitation filter of 533–558 nm and an emission filter of 570–640 nm were used.

The aim of this study was to develop and test an easy, quick and affordable method based on the extraction and quantification of another pigment, the phycocyanin (PC), to estimate moss-associated cyanobacteria quantity. This approach is inspired by a method commonly used to monitor cyanobacteria blooms in lakes for the last 20 years (Seppälä et al., 2007; McQuaid et al., 2011). PC is a photosynthesis pigment produced by cyanobacteria and located in phycobilisome structures in the thylakoid membrane (MacColl, 1998). This pigment is already commonly used to observe and count moss-associated cyanobacteria by epifluorescence microscopy (Figure 1). Phycocyanin has been reported as the most abundant pigment among phycobiliproteins, the major light-harvesting pigments, and can account for 20% of the total proteins in the cyanobacteria dry mass (de Marsac, 1977; Stanier and Cohen-Bazire, 1977). Choosing phycocyanin as a quantitative marker has several advantages over other cyanobacterial pigments. First, phycocyanin is produced only by cyanobacteria and two groups of algae (the cryptophytes and the rhodophytes; Kirk, 1994) and is not found in moss, unlike some chlorophylls and carotenoids (such as echinenone, Czygan, 1981). Then, it is water-soluble and can be easily measured by fluorimetry. Fluorescence spectroscopy is a very sensitive technique and is less laborious than HPLC based methods, which require chromatographic separation, that are commonly used to quantify other cyanobacteria marker pigments like chlorophyll a or echinenone (Schalles and Yacobi, 2000; Poza-Carrión et al., 2001; Schlüter et al., 2004; Seppälä et al., 2007; Arróniz-Crespo et al., 2014). Phycocyanin measurements have been extensively used to quantify cyanobacteria in pure cultures (Herrera et al., 1989; Lee et al., 2017; Basheva et al., 2018; Piron et al., 2019) and water samples (Izydorczyk et al., 2005; Cotterill et al., 2019; Cegłowska et al., 2020) but, to our knowledge, it has never been applied to moss samples. We proposed that phycocyanin quantification could be a reliable proxy to estimate moss-associated cyanobacteria quantity. To evaluate if this new approach can be extensively used, we tested four important analytical performance parameters. We first performed the analytical calibration of the method by evaluating (i) cellular phycocyanin linearity in pure cultures of five cyanobacterial strains, (ii) the range of applicability of the method applied to moss samples, and (iii) the detection and quantification limits of phycocyanin. Then, we studied (iv) the accuracy of the method by measuring three performance parameters: phycocyanin apparent recovery assessed using both phycocyanin standard and cyanobacteria culture spikes on moss, matrix effects and repeatability. Then, we applied the method directly on two feather moss species that are dominating in the eastern Canadian boreal forest, *Pleurozium schreberi* (Brid.) Mitt. and *Ptilium crista-castrensis* (Hedw.) De Not. (Harper et al., 2003). Finally, because the quantity of cyanobacteria associated with feather moss has been reported to be correlated with BNF activity in *P. schreberi*, using microscopic counting (DeLuca et al., 2007) and qPCR (Warshan et al., 2016), we also assessed the relationship between phycocyanin and BNF in moss samples to evaluate if the relationship observed with other quantification methods was similar when using phycocyanin measurements.

## Materials and Methods

### Cyanobacterial Strains Selection and Culture Conditions

To evaluate phycocyanin linearity and phycocyanin apparent recovery (see analytical performance parameters assessment section), we cultivated cyanobacteria in defined laboratory conditions.

To test phycocyanin linearity in cyanobacteria cultures, we used five cyanobacterial strains belonging to the *Nostoc* genus, isolated from the feather moss *P. crista-castrensis* and *Peltigera* cyanolichens collected in Quebec, Canada, and Iceland (Table 1). We selected *Nostoc* sp. strains because they were found to be commonly associated with boreal feather moss (DeLuca et al., 2002; Ininbergs et al., 2011). To determine phycocyanin apparent recovery in moss samples, we spiked moss with *Anabaena variabilis* (ATCC 29413) cells. *Anabaena variabilis* is an aquatic cyanobacteria often assessed during bloom monitoring (Li et al., 2016). We used *A. variabilis* for the phycocyanin apparent recovery experiments because of its high phycocyanin cell content (10 times higher than *Nostoc* sp. in average), which allows to having a higher phycocyanin signal using less cyanobacteria cells. Moreover, in cultures, *A. variabilis* produced significantly less biofilm than *Nostoc* sp. strains, allowing for a more accurate cell harvesting by pipetting. All cyanobacteria strains were grown on a liquid N-free BG11_0_ medium (Rippka et al., 1979) at 22°C, under continuous white fluorescent light tubes (T8 bulb, Sylvania Gro-Lux) at 30 μmol.m^−2^.s^−1^ and without agitation. Cyanobacteria cells were harvested at the beginning of the stationary growth phase (20 days).

**Table 1:** Cyanobacterial strains used in this study.

Species	Strain	Isolation	Country of Origin
*Nostoc* sp.	210A	*Peltigera membranacea*	Iceland
*Nostoc* sp.	213	*Peltigera membranacea*	Iceland
*Nostoc* sp.	232	*Peltigera membranacea*	Iceland
*Nostoc* sp.	MR100	*Ptilium crista-castrensis*	Canada
*Nostoc* sp.	MR101	*Peltigera* sp.	Canada
*Anabaena variabilis*	ATCC 29413	Freshwater	United States

### Moss Sampling

Feather moss samples were collected for both the evaluation of analytical performance parameters (see section below) and to test the relationship between BNF and phycocyanin quantity.

*Pleurozium schreberi* and *Ptilium crista-castrensis* were collected on four boreal forest sites along a 500-km latitudinal transect in Quebec, Canada in June and September 2019. All sites are located between N47° and N51°, in the black spruce forest bioclimatic zone. As environmental parameters are known for affecting cyanobacteria BNF, sampling sites are spread along a latitudinal gradient of temperature, moisture, and atmospheric deposition to examine the relationship between phycocyanin and BNF for moss samples displaying potentially contrasting BNF activities. Samples were collected in plastic bags and kept at 4°C in the dark until being processed, within 2 weeks after collection.

### Phycocyanin Extraction and Quantification Procedure

**Figure 2:**
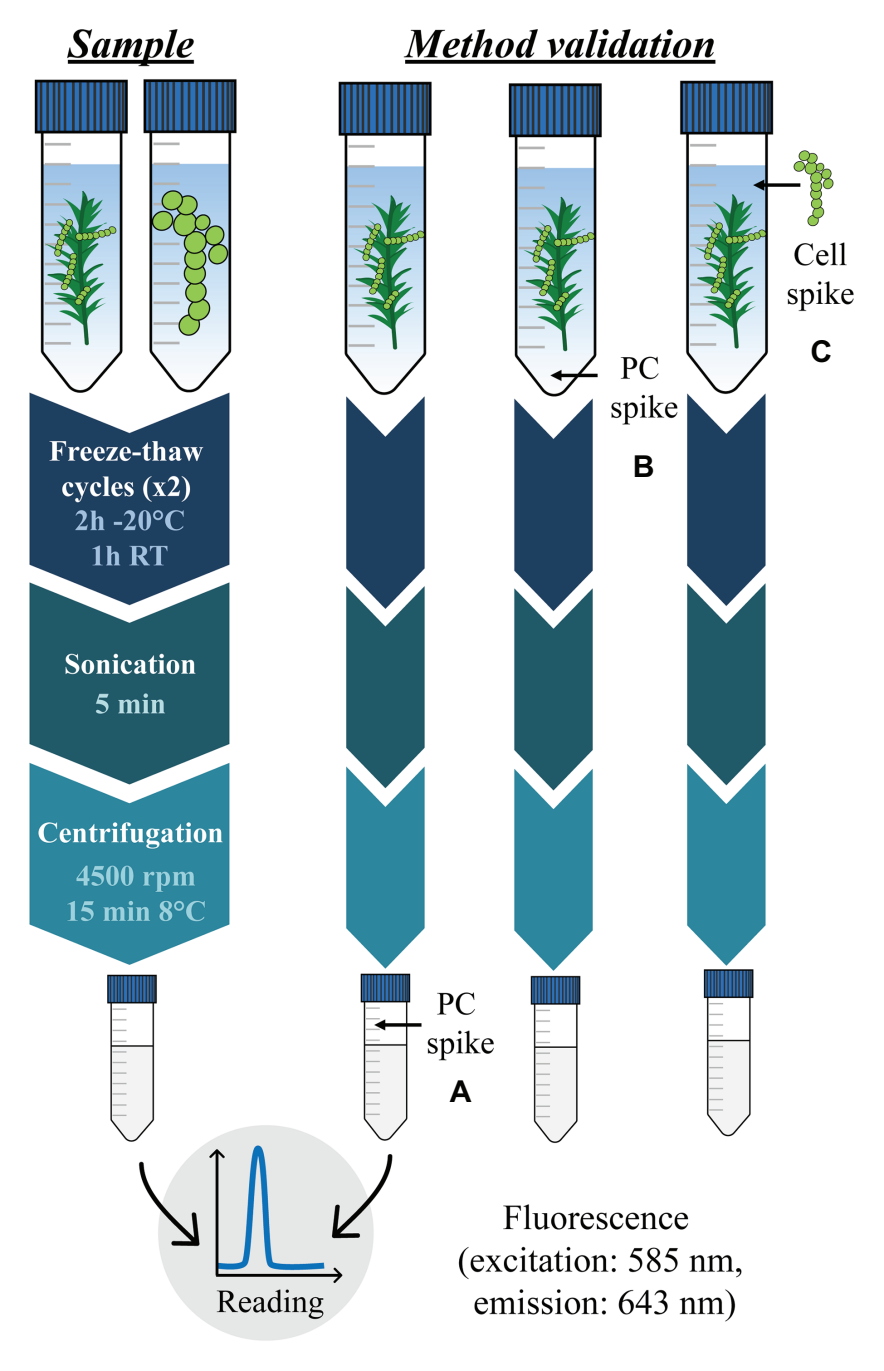
Phycocyanin (PC) extraction and quantification procedure for cyanobacteria in cultures and moss samples (left) and method validation process (right). Capital letters in brackets refer to three analytical performance parameters evaluated for the method validation. **(A)** corresponds to matrix effects, **(B)** corresponds to standard phycocyanin apparent recovery, and **(C)** corresponds to cellular phycocyanin apparent recovery.

The phycocyanin extraction method is divided in four consecutive important steps: (i) sample dilution in sodium phosphate buffer, (ii) disruption of cyanobacteria cell membranes, (iii) phycocyanin solubilization in sodium phosphate buffer, and (iv) quantification by fluorescence of the solubilized phycocyanin. These steps are described in detail in the following section and in Figure 2.

**Figure 3:**
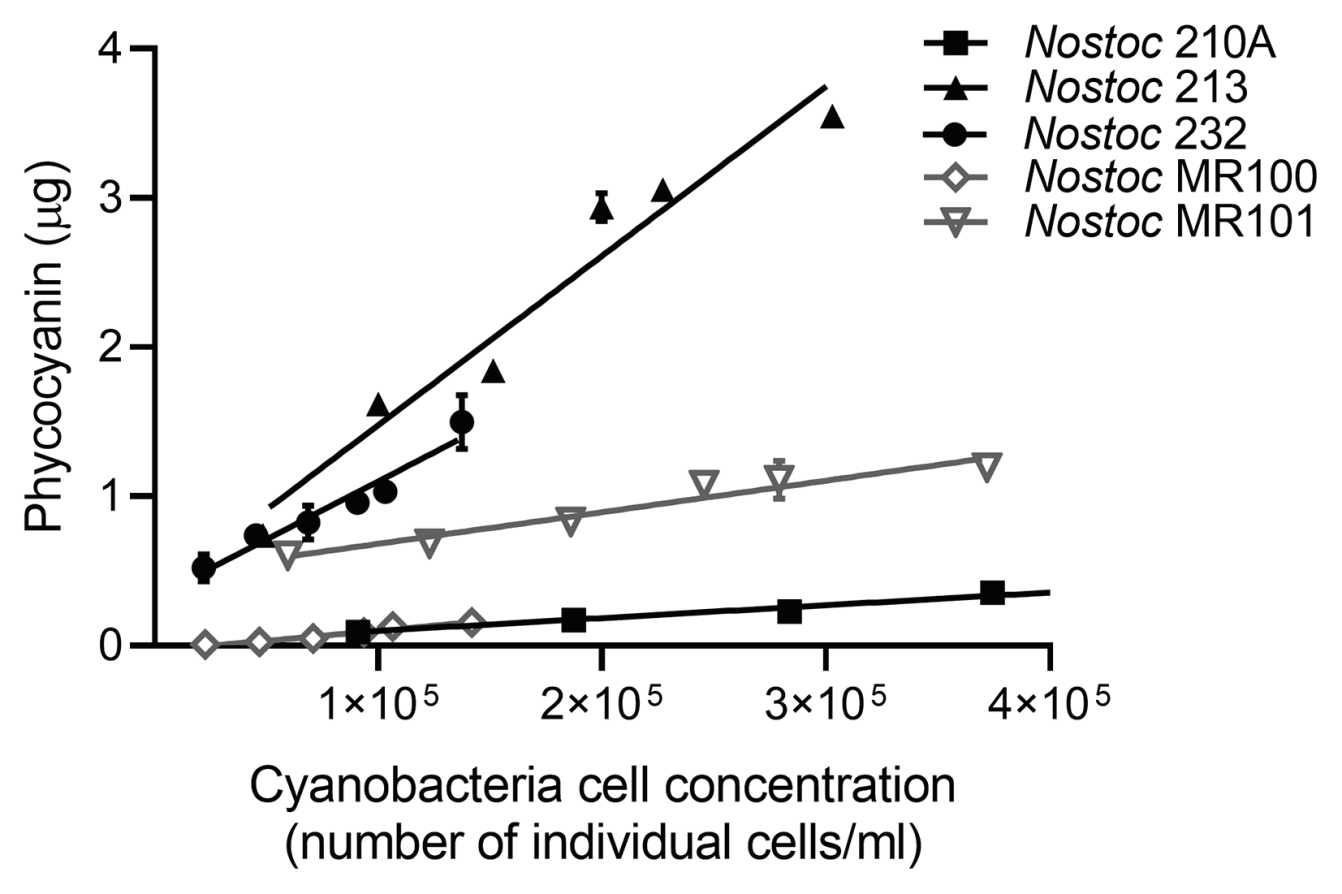
Phycocyanin mass mean ± SD (*n* = 3) linearity in cyanobacteria *Nostoc* sp. pure cultures. Straight lines represent the linear regression between phycocyanin mass and the number of individual cyanobacteria cell per ml of culture, for each cyanobacterial strain. For all strains, *R*^2^ > 0.93 and *p* < 0.002.

For phycocyanin extraction of cyanobacteria in cultures, we performed a series of dilution of cell suspensions for each strain. Between 2.5 and 12.5 ml of culture were harvested and diluted in sterilized sodium phosphate buffer (0.025 M, pH 7) to reach a final volume of 15 ml. Cyanobacteria cell concentrations, corresponding to the number of individual cells per ml of culture, are presented in Figure 3. For phycocyanin extraction of cyanobacteria living on feather moss, samples were oven-dried at 35°C for 4 h and milled with a blender. Then, 0.15–0.20 g of moss sample were placed in 50 ml sterile tubes with 15 ml of sterilized sodium phosphate buffer (0.025 M, pH 7; Sarada et al., 1999; Furuki et al., 2003; Horváth et al., 2013) prepared by mixing 2.99 g of Na_2_HPO_4_ • 2H_2_O and 1.91 g of NaH_2_PO_4_ • 2H_2_O in 1 L of Milli-Q water. Phycocyanin extraction procedure was then performed similarly for cyanobacteria cells and moss samples. All samples (i.e., cyanobacteria cultures or moss in sodium phosphate buffer) were homogenized by vortexing and shaking manually for 10 s. To achieve cyanobacteria cell membrane disruption, samples were subjected to two freeze-thaw cycles (2 h at −20°C followed by 1 h at room temperature), mixed by vortexing for 10 s between cycles (Lawrenz et al., 2011; Horváth et al., 2013) and sonicated for 5 min in an ultrasound bath. Then, samples were centrifuged at 3,400 × *g* at 8°C for 15 min. Supernatants were transferred to 15 ml tubes and stored at −80°C until analysis. In these conditions, extracted phycocyanin can be stored for up to 6 months without noticeable degradation (Lawrenz et al., 2011). Phycocyanin was quantified by spectrofluorometry (excitation at 585 nm and emission at 643 nm; Seppälä et al., 2007) on a QuantaMaster 400 Phosphorimeter (PTI) using a commercial standard (C-Phycocyanin, Sigma-Aldrich). All tubes were covered by aluminum foil during the entire procedure to limit photodegradation. After phycocyanin extraction, moss samples were oven-dried at 50°C and weighted. Results were expressed in μg of phycocyanin (mass) or in μg of phycocyanin per g of moss (concentration). All phycocyanin concentrations measured were above detection and quantification limits.

### Analytical Performance Parameters Assessment

To assess the reliability of the method and evaluate if it can be applied to moss, we tested different analytical performance parameters.

### Analytical Calibration of the Method

We first tested if phycocyanin mass was linear within a large range of cyanobacteria cell quantity by measuring phycocyanin in cyanobacteria pure cultures. For each cyanobacteria strain, cultivated cells were harvested and diluted in triplicates with sodium phosphate buffer, prior to phycocyanin extraction. Both procedures are described in the section above. The number of vegetative cells in cyanobacteria cultures was measured using a counting chamber (Petroff-Hausser counting chamber, Hausser Scientific). Briefly, 2 μl of each culture were placed in the counting chamber and individual cells were counted using a bright field microscope (Motic BA210). The counting procedure was repeated five times per strain. Cell number per ml of culture was calculated according to the formula provided by the manufacturer and final cyanobacteria cell concentrations were attributed to each culture dilution. All the following analytical performance parameters were measured for both moss species *P.-castrensis* and *P. schreberi*.

We determined the range of applicability of the method (i.e., the range of mass of moss within which phycocyanin quantity is linear) by extracting phycocyanin from six different mass of moss, comprised between 0.25 and 1.25 g. The exact same moss sample was used for this experiment and three technical replicates per mass of moss were performed.

Limit of detection (LOD) and limit of quantification (LOQ) of phycocyanin were respectively calculated by adding 3 × SD and 10 × SD to the average signal measured in analytical blanks.

### Accuracy of the Method

Then, we determined the accuracy of the method (i.e., estimation of the total error impacting the method; Raposo and Ibelli-Bianco, 2020) composed of two elements, the trueness (i.e., evaluation of the systematic error), and the precision (i.e., evaluation of the random error). Trueness was examined by measuring phycocyanin apparent recovery and matrix effects. Precision was evaluated through testing the repeatability. Phycocyanin apparent recovery is defined as the ratio between observed values (i.e., measured phycocyanin) and reference values (i.e., estimated added phycocyanin). We determined (i) the standard phycocyanin apparent recovery by adding a C-phycocyanin commercial standard (Sigma-Aldrich) on moss and (ii) the cyanobacteria cellular phycocyanin apparent recovery by adding *A. variabilis* cells on moss. Cellular phycocyanin linearity in *A. variabilis* culture was checked prior to this experiment (Supplementary Figure S1) and both standard and cellular phycocyanin apparent recoveries were determined using four replicates of the same moss sample. Standard phycocyanin (Figure 2B) and cellular phycocyanin apparent recoveries (Figure 2C) were respectively determined by spiking phycocyanin standard at 0.5×, 2×, and 15× the average phycocyanin content in moss and by spiking between 2 and 12 ± 1.5 × 10^6^ *A. variabilis* cells, which corresponds to additions of 14–84 μg of phycocyanin (Supplementary Figure S1), on 0.60 ± 0.1 g of moss.

Matrix effects (i.e., components present in samples potentially affecting phycocyanin quantification; Raposo and Barceló, 2020) were evaluated by adding a C-phycocyanin commercial standard to samples of phycocyanin extracted from moss (Figure 2A). Standard additions were performed on five replicates at 0.5×, 2×, and 15× the average phycocyanin content in moss.

Finally, the repeatability of the method (i.e., the closeness of phycocyanin results obtained by analyzing the same sample using the same procedure and under similar conditions; Thompson and Wood, 1993) was assessed by extracting phycocyanin from six replicates of the same moss sample for 3 consecutive days. Analytical blanks were performed and analyzed for each set of experiment.

### Nitrogen Fixation Measurements

Green parts (i.e., photosynthetic parts of the moss shoots containing the cyanobacteria colonies) of *P. schreberi* and *P.-castrensis* shoots were hydrated with deionized water to homogenize their hydration state and placed into 250 ml glass jars. This experiment was performed in four replicates for all moss species, site, and date of collection. Samples were acclimated for 5 days in a growth chamber (18°C; 16 h light, 8 h dark) and moss-associated bacteria BNF was assessed using ARA (Hardy et al., 1968). Acetylene gas was produced by adding 25 ml of H_2_O to 5 g of CaC_2_ (Acros Organics) in Tedlar® gas sampling bags (Sigma-Aldrich). Moss samples were incubated with 20% of acetylene for 24 h at 18°C under continuous light. Ethylene production was measured on a gas chromatograph (Shimadzu 8A with an FID detector and a Supelco column 01282011). Following ARA, moss samples were processed for phycocyanin extraction, therefore moss dry mass could not be directly determined to standardize ARA. Moss dry mass was calculated by measuring the average moss water content by surface unit on four replicates per species and per sampling site. The estimated moss dry mass used for the ARA was comprised between 1.8 and 3 g per replicate.

### Statistical Analysis

The test of Kruskal and Wallis (1952) followed by the Dunn’s (1965) *post hoc* test were used to examine the effects of moss species and month of collection on phycocyanin concentrations. Normality was tested with the Shapiro-Wilk test (Royston, 1995) and linear regression outliers were checked using the test of Grubbs (1969). Linear regressions and statistical tests were respectively performed using GraphPad Prism (version 8.0.2) and R (version 3.4.3, R Core Team, 2017) with the Stats package. Statistically significant differences were accepted for value of *p* < 0.05.

## Results and Discussion

### Phycocyanin Extraction Method Characterization and Validation

**Figure 4:**
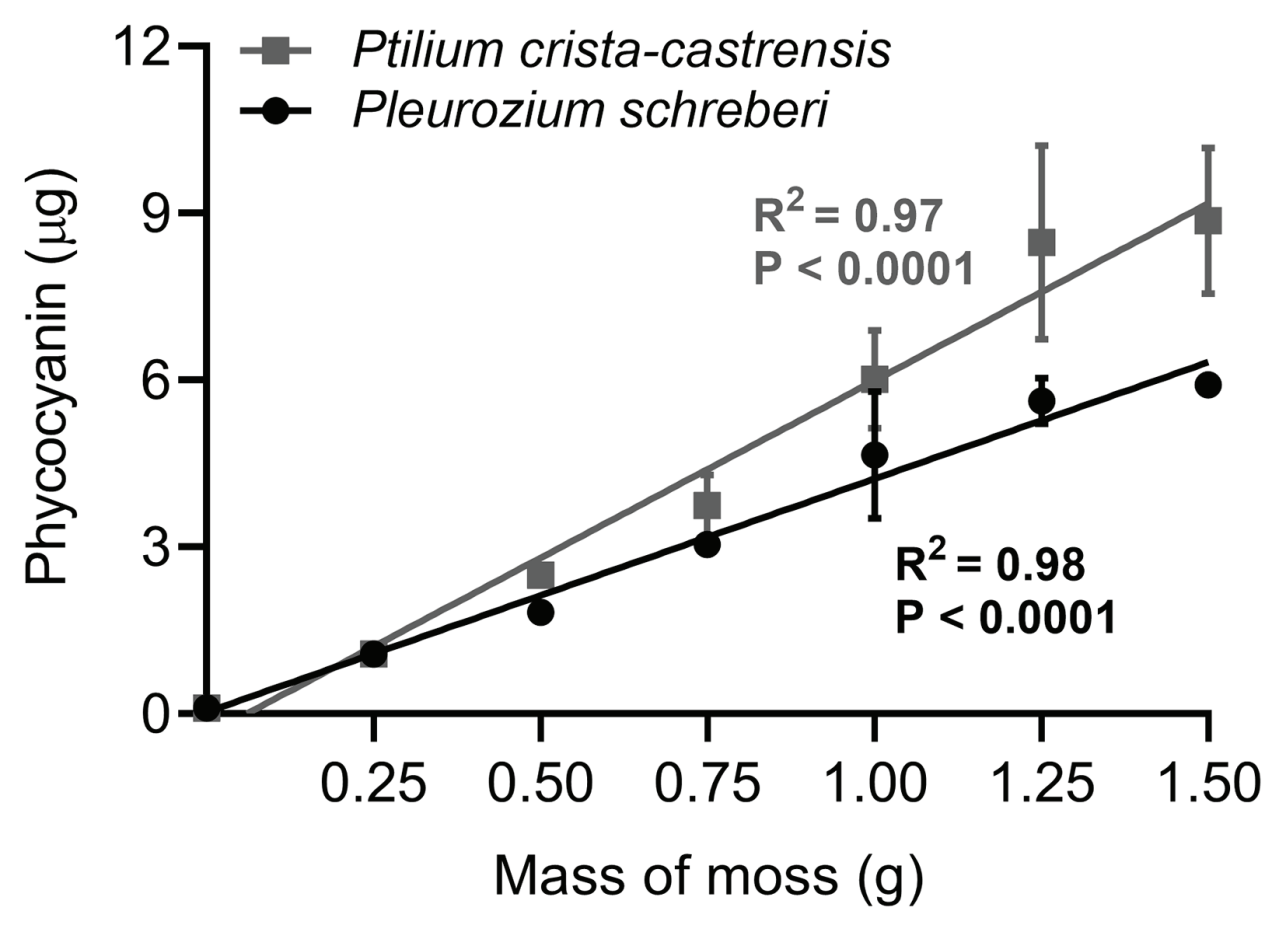
Phycocyanin mass mean ± SD (*n* = 3) linearity in *P. crista-castrensis* (gray squares) and *Pleurozium schreberi* (black circles) feather moss. Straight lines represent the linear regression between phycocyanin and moss masses, for each moss species. All replicates are from a same moss sample of each species, that were acclimated for 5 days in a growth chamber (30°C, 16 h light, 8 h dark) before being processed.

All the analytical performance parameters measured in this study and used to validate the method are presented in Table 2. We first tested phycocyanin linearity for a wide range of cyanobacteria cell densities in pure liquid cultures of five strains of *Nostoc* sp. extracted from the boreal moss species *P.-castrensis*, and *Peltigera* cyanolichens (Table 1). For all strains, phycocyanin mass was strongly correlated with cell density (*R*^2^ > 0.93, Figure 3), showing that phycocyanin is a reliable proxy for quantifying cyanobacteria cells, even over a large range of cell counts. However, significant differences in phycocyanin cellular concentration were observed between strains. Using data from Figure 3, we calculated phycocyanin cellular concentration for each cyanobacteria strain. *Nostoc* 232 achieved the highest phycocyanin cellular concentration with an average of 1.18 ± 0.39 × 10^−6^ μg.cell^−1^, followed by *Nostoc* 213 (9.24 ± 1.1 × 10^−7^ μg.cell^−1^), *Nostoc* MR101 (3.58 ± 1.6 × 10^−7^ μg.cell^−1^), *Nostoc* MR100 (8.4 ± 1.5 × 10^−8^ μg.cell^−1^), and *Nostoc* 210A (6.73 ± 9.6 × 10^−9^ μg.cell^−1^). Then, we tested the range of applicability of the method for moss samples by examining phycocyanin linearity for a large range of moss masses. For both moss species, phycocyanin and moss masses were strongly correlated (*R*^2^ = 0.97 for *P. crista-castrensis* and *R*^2^ = 0.98 for *P. schreberi*, Figure 4). This shows that phycocyanin extraction efficiency is not dependent of moss mass in the tested range of 0.25–1.50 g.

**Table 2:** Analytical performance parameters tested in this study.

	Apparent recovery ± SE (%)	Matrix effects ± SE (%)	Repeatability RSD (%)	Limit of detection (μg.L^−1^)	Limit of quantification (μg.L^−1^)	Range of applicability (g of moss)
*Ptilium crista-castrensis*	45*^a^* ± 7.6 39*^b^* ± 3.4	105.8 ± 10.6	11.6	3.1	4.3	0.25–1.50
*Pleurozium schreberi*	69*^a^* ± 16.8 50.8*^b^* ± 4.2	100.7 ± 4.5	12.4			

a The apparent recovery after C-phycocyanin standard spikes.

b The apparent recovery after *Anabaena variabilis* cells spikes on moss.

Using analytical blanks, we found that phycocyanin LOD and LOQ were 3.1 and 4.3 μg.L^−1^ respectively.

We also determined standard phycocyanin and cellular phycocyanin apparent recoveries by spiking a commercial standard and *A. variabilis* cells on moss. The average standard apparent recoveries were 45 ± 7.6% for *P. crista-castrensis* and 69 ± 16.8% for *P. schreberi*. Average cellular apparent recoveries were lower for both moss species, we found 39 ± 3.4% for *P. crista-castrensis* and 50.8 ± 4.2% for *P. schreberi*. No significant matrix effects were observed with an average recovery of spiked C-phycocyanin standard in moss extract of 105.8 ± 10.6% for *P. crista-castrensis* and 100.7 ± 4.5% for *P. schreberi*. Phycocyanin recoveries were similar for the three spike concentrations tested. The repeatability (i.e., inter-day precision) was satisfactory with average relative standard deviations (RSD) of 12.4 and 11.6% for *P. schreberi* and *P. crista-castrensis*, respectively.

Kissoudi et al. (2018) quantified phycocyanin extracted from a cyanobacteria culture by HPLC and found greatly higher LOD and LOQ (670 and 2000 μg.L^−1^ respectively), showing that spectrofluorometry is a more suitable quantification technique for phycocyanin. In this study, all samples (cyanobacteria cultures and mosses) had phycocyanin concentration above those limits. Phycocyanin apparent recoveries in moss found in this study are close to the average apparent recovery reported in pure cultures (50–60%, Tavanandi et al., 2018) but optimized methods can reach 90–92% (Kissoudi et al., 2018; Prates et al., 2018; Tavanandi et al., 2018). Phycocyanin concentration measured in cyanobacteria cultures depends greatly on the extraction process (e.g., solvent, extraction time, cell wall disruption technique; Abalde et al., 1998; Reis et al., 1998). A critical step for phycocyanin extraction is the cell membrane disruption step, which, when incomplete, can affect phycocyanin recovery (Stewart and Farmer, 1984). The thermal insulation properties of the moss (Bakatovich and Gaspar, 2019), for example, could prevent an efficient cyanobacteria cell wall disruption. Moreover, we found significantly lower apparent recoveries for cyanobacteria cells addition compared to phycocyanin standard addition (Table 2) suggesting that cyanobacteria cellular structures can impact phycocyanin extraction. Cyanobacteria cellular lyse efficiency (cellular apparent recovery/standard apparent recovery, C/B, Figure 2) achieved 86.7% for *P. crista-castrensis* and 73.6% for *P. schreberi*, which confirms that cell wall breaking was not optimal after the phycocyanin extraction procedure. Chittapun et al. (2020) also showed that several species of cyanobacteria had specific cell wall structures which necessitated to using different disruption techniques for each species to obtain an optimal phycocyanin efficiency. Besides the cell wall disruption efficiency, the lower apparent recoveries of cellular phycocyanin could also be explained by the presence of biofilm in cyanobacteria culture, preventing accurate cell counting and pipetting. In addition, apparent recovery might be affected by possible mechanisms of phycocyanin adsorption on moss cell walls after release from cyanobacteria cells. Phycocyanin degradation by temperature (Antelo et al., 2008) and light (Jespersen et al., 2005) over time are potential causes of extraction efficiency loss that we considered minimal in our experiments because moss samples were always kept at cool temperature and in the dark during the extraction. Repeatability reported here are also similar to values reported in literature for pure culture (Kissoudi et al., 2018; Prates et al., 2018). Thus, our results show that the efficiency of cyanobacteria cell wall disruption is the principal factor that could affect the quality of phycocyanin measurements but that, overall, phycocyanin can be accurately and reliably quantified for large cyanobacterial cell density and moss mass ranges.

### Advantages and Limits of the Method

Phycocyanin extraction is a quick, simple, and affordable method to assess cyanobacteria quantity living on moss. This method allows estimating cyanobacteria abundance on a great number of samples in limited time and effort (approximately, the extraction procedure takes 6 h30 and the quantification takes 2 h30 for 50 samples). Because of the heterogeneity in cyanobacteria density within and between moss shoots, many leaves and/or shoots need to be processed to achieve a reasonable estimate of cyanobacteria number per shoots using microscopic counting. With phycocyanin extraction, cyanobacteria quantity can be estimated using many moss shoots (or subsamples of large amounts of homogenized shoots), allowing for an efficient integration of heterogeneity and thus for more reliable estimates of the average number of cyanobacteria. Lastly, phycocyanin extraction is also more environmentally friendly than other pigment extraction techniques because it does not require harmful organic solvents like acetone or hexane (Papadaki et al., 2017). Phycocyanin extraction in a water-based solvent also has the advantage to limit the co-extraction of other pigments poorly soluble in water, such as chlorophylls, that could interfere with phycocyanin quantification.

However, phycocyanin extraction also has some limitations. Differences in cellular phycocyanin concentration between cyanobacteria strains have been reported in pure cultures (this study, Rippka et al., 1979; Santiago-Santos et al., 2004; Chittapun et al., 2020). This can be explained by variable phycocyanin production per cell, as well as differences in extraction efficiencies due to cell wall thickness and biofilm production, differing among cyanobacteria strains (Chittapun et al., 2020). The composition of cyanobacteria communities colonizing moss can vary with environmental conditions, moss species and time of sampling during the growth season (Ininbergs et al., 2011; Rousk et al., 2013; Warshan et al., 2016). In addition to the inherent variation of phycocyanin quantity among cyanobacteria species, growth phase (McQuaid et al., 2011; Chang et al., 2012) and growth conditions (e.g., culture medium, light, temperature, and nutrient stress) has been shown to affect phycocyanin production (De Morais et al., 2018). For example, N limited availability or high photoperiod can decrease phycocyanin concentration in cyanobacteria cultures (Sloth et al., 2006; Ürek and Tarhan, 2012; Prates et al., 2018). Thus, comparison of phycocyanin data, used as cyanobacteria cell number estimates, from sites characterized by contrasted environmental conditions and from moss samples with different cyanobacteria communities should be made with caution. These limitations, due to species-specific phycocyanin cell content varying with environmental conditions, were also reported to impact the quantification of cyanobacteria using phycocyanin in aquatic ecosystems (Seppälä et al., 2007). Calibration of the method (e.g., phycocyanin linearity and apparent recovery) using strains isolated from moss species and sampling sites of interest could alleviate this potential bias.

### Phycocyanin Concentration and Nitrogen Fixation in Boreal Feather Moss

**Figure 5:**
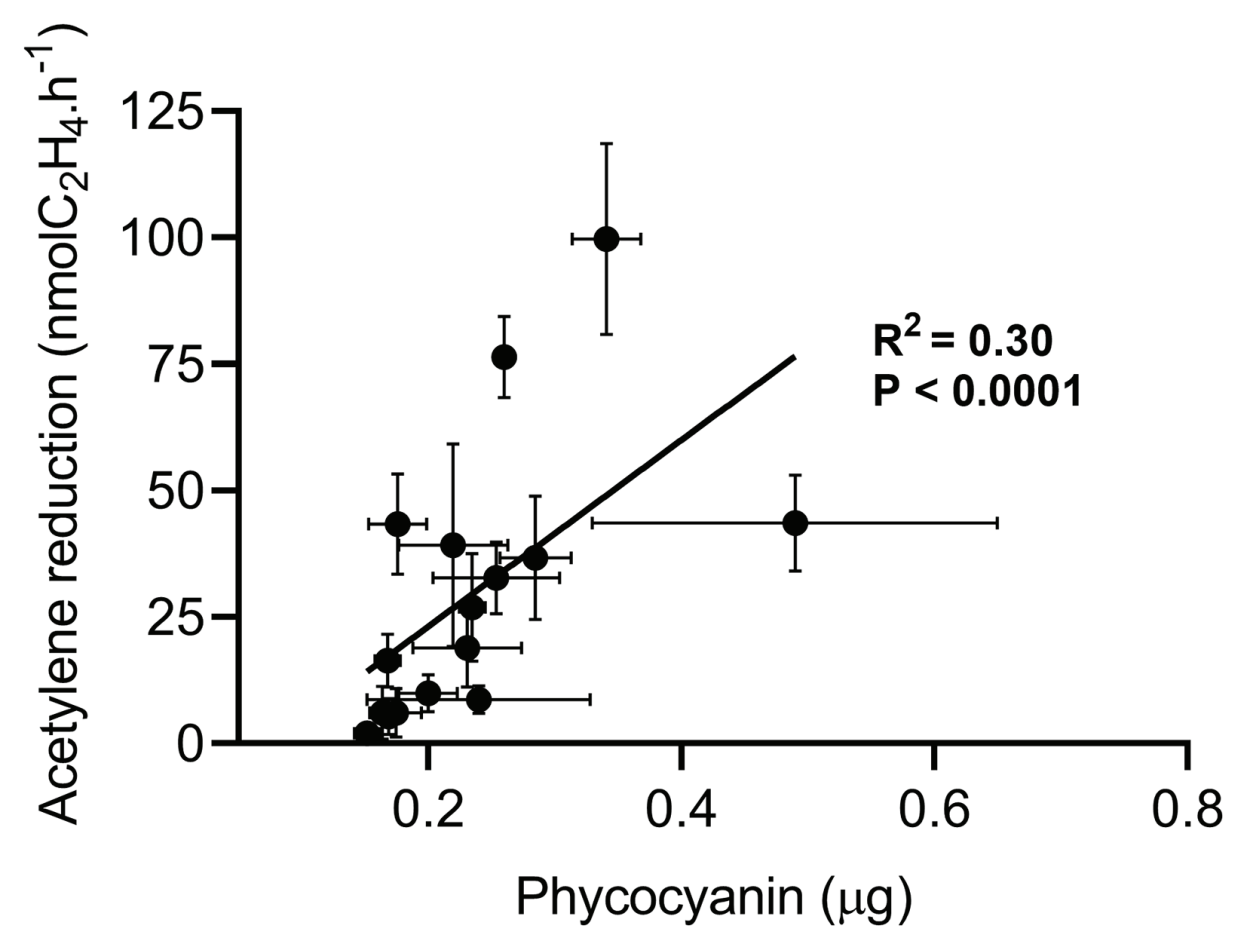
Linear regression between acetylene reduction mean ± SD (*n* = 4) and phycocyanin mass mean ± SD (*n* = 3) of feather moss *P. crista-castrensis* and *P. schreberi* collected in Quebec, Canada in June and September 2019. The linear regression regroups a total of 64 observations for acetylene reduction and 48 observations for phycocyanin measurements. All samples were acclimated for 5 days in a growth chamber (18°C; 16 h light, 8 h dark) before being processed for acetylene reduction and phycocyanin, consecutively.

Direct comparison of phycocyanin extraction with other cyanobacteria quantification methods is delicate and would be poorly informative since each method (e.g., microscope counting, qPCR, and echinenone extraction) has its own flaws. Thus, we decided to evaluate how phycocyanin extraction compares to other methods using an independent measurable: the BNF. Several studies suggested that in low N deposition areas, mosses control the colonization of cyanobacteria based on their N demand for growth (DeLuca et al., 2007; Gundale et al., 2011; Rousk et al., 2013). For *Pleurozium schreberi* collected in Scandinavia, BNF activity was showed to be closely related to cyanobacteria quantity measured by microscopic counting (DeLuca et al., 2007; Rousk et al., 2013; Warshan et al., 2016). Another study from Chile reported a similar linear relationship between BNF and moss-associated cyanobacteria quantity using echinenone, a pigment produced by cyanobacteria (Arróniz-Crespo et al., 2014). Thus, assuming that phycocyanin is a reliable proxy for quantifying cyanobacteria, phycocyanin, and BNF should be correlated in moss samples collected in low N deposition (< 3 kg.ha^−1^.yr.^−1^) forest sites in Eastern Canada. We measured phycocyanin and BNF of *P. schreberi* and *P. crista-castrensis* from four sites at the beginning (June) and the end (September) of the growth season. Phycocyanin concentrations varied between 0.45–1.26 μg.g^−1^ in June and 0.56–0.96 μg.g^−1^ in September for *P. crista-castrensis* and 0.42–0.48 μg.g^−1^ in June and 0.61–0.88 μg.g^−1^ in September for *P. schreberi* (Supplementary Table S1). We found no significant effect of the sampling month on phycocyanin concentration (value of *p* = 0.075) but we report a significant effect of the moss species on phycocyanin content (value of *p* = 3.3 × 10^−4^) confirming the need for testing and calibrating the phycocyanin extraction method on the chosen moss species, as discussed earlier. Phycocyanin concentrations of both moss species collected in June and September were positively correlated with BNF activity (*R*^2^ = 0.30, Figure 5). We found a correlation coefficient relatively similar to those reported by Arróniz-Crespo et al. (2014) using echinenone quantification (*R*^2^ = 0.44) and DeLuca et al. (2007) using microscopic counting (*R*^2^ = 0.58).

This result suggests that phycocyanin extraction is a valuable semi-quantitative method, allowing for the rapid assessment of cyanobacteria abundance living on moss. Using phycocyanin for the quantification of cyanobacteria in moss suffers from the same major limitation as the quantification of cyanobacteria in aquatic ecosystems: cellular phycocyanin concentrations vary among species and strains. For moss, as in aquatic systems, evaluating the effects of environmental factors on the number of cyanobacteria requires the rapid analysis of large numbers of samples. Phycocyanin extraction allows such rapid and high throughput analysis in aquatic systems. While further studies on other moss species, sites, and environmental conditions, are required to validate the potential of phycocyanin quantification as a proxy of cyanobacteria quantity, our results strongly suggest that phycocyanin also represents an easy, rapid, and affordable way to evaluate moss-associated cyanobacteria quantity. Phycocyanin quantification can guide the use of more costly and time-consuming complementary methods to estimate cyanobacteria abundance (e.g., qPCR). Further work should be done on better characterizing cyanobacteria communities associated with feather moss to improve our understanding of its impact on cyanobacteria quantification estimated with phycocyanin measurements.

## Data Availability Statement

The original contributions presented in the study are included in the article/Supplementary Material, further inquiries can be directed to the corresponding author.

## Author Contributions

MR, RD, and J-PB designed the study and the experiments. MR performed all the experiments and data analyses and wrote the manuscript. RD and J-PB revised the manuscript. All authors contributed to the article and approved the submitted version.

### Conflict of Interest

The authors declare that the research was conducted in the absence of any commercial or financial relationships that could be construed as a potential conflict of interest.

## References

[ref1] Abalde J. Betancourt L. Torres E. Cid A. Barwell C. (1998). Purification and characterization of phycocyanin from the marine cyanobacterium *Synechococcus* sp. IO9201. Plant Sci. 136, 109–120. 10.1016/S0168-9452(98)00113-7

[ref2] Antelo F. S. Costa J. A. V. Kalil S. J. (2008). Thermal degradation kinetics of the phycocyanin from *Spirulina platensis*. Biochem. Eng. J. 41, 43–47. 10.1016/j.bej.2008.03.012

[ref3] Arróniz-Crespo M. Pérez-Ortega S. De Los Ríos A. Green T. G. A. Ochoa-Hueso R. Casermeiro M. Á.. (2014). Bryophyte-cyanobacteria associations during primary succession in recently deglaciated areas of Tierra del Fuego (Chile). PLoS One 9:e96081. 10.1371/journal.pone.0096081, PMID: 24819926 PMC4018330

[ref4] Bakatovich A. Gaspar F. (2019). Composite material for thermal insulation based on moss raw material. Constr. Build. Mater. 228:116699. 10.1016/j.conbuildmat.2019.116699

[ref5] Basheva D. Moten D. Stoyanov P. Belkinova D. Mladenov R. Teneva I. (2018). Content of phycoerythrin, phycocyanin, alophycocyanin and phycoerythrocyanin in some cyanobacterial strains: applications. Eng. Life Sci. 18, 861–866. 10.1002/elsc.201800035, PMID: 32624879 PMC6999198

[ref6] Cegłowska M. Toruńska-Sitarz A. Stoń-Egiert J. Mazur-Marzec H. Kosakowska A. (2020). Characteristics of cyanobacterium *Pseudanabaena galeata* CCNP1313 from the Baltic Sea. Algal Res. 47:101861. 10.1016/j.algal.2020.101861

[ref7] Chang D. W. Hobson P. Burch M. Lin T. F. (2012). Measurement of cyanobacteria using in-vivo fluoroscopy-effect of cyanobacterial species, pigments, and colonies. Water Res. 46, 5037–5048. 10.1016/j.watres.2012.06.050, PMID: 22824675

[ref8] Chittapun S. Jonjaroen V. Khumrangsee K. Charoenrat T. (2020). C-phycocyanin extraction from two freshwater cyanobacteria by freeze thaw and pulsed electric field techniques to improve extraction efficiency and purity. Algal Res. 46:101789. 10.1016/j.algal.2020.101789

[ref9] Cotterill V. Hamilton D. P. Puddick J. Suren A. Wood S. A. (2019). Phycocyanin sensors as an early warning system for cyanobacteria blooms concentrations: a case study in the Rotorua lakes. N. Z. J. Mar. Freshw. Res. 53, 555–570. 10.1080/00288330.2019.1617322

[ref10] Czygan F. -C. (1981). Echinenone (ꞵ,ꞵ-caroten-4-one) from the gametophyte of *Polytrichum formosum* (Bryophyta, Musci). Z. Pflanzenphysiol. 103, 169–171. 10.1016/s0044-328x(81)80147-x

[ref11] de Marsac N. T. (1977). Occurrence and nature of chromatic adaptation in cyanobacteria. J. Bacteriol. 130, 82–91. 10.1128/jb.130.1.82-91.1977, PMID: 856789 PMC235176

[ref12] De Morais M. G. Da Fontoura Prates D. Moreira J. B. Duarte J. H. Costa J. A. V. (2018). Phycocyanin from microalgae: properties, extraction and purification, with some recent applications. Ind. Biotechnol. 14, 30–37. 10.1089/ind.2017.0009

[ref13] DeLuca T. H. Boisvenue C. (2012). Boreal forest soil carbon: distribution, function and modelling. Forestry 85, 161–184. 10.1093/forestry/cps003

[ref14] DeLuca T. H. Zackrisson O. Gentili F. Sellstedt A. Nilsson M. C. (2007). Ecosystem controls on nitrogen fixation in boreal feather moss communities. Oecologia 152, 121–130. 10.1007/s00442-006-0626-6, PMID: 17219131

[ref15] DeLuca T. H. Zackrisson O. Nilsson M. C. Sellstedt A. (2002). Quantifying nitrogen-fixation in feather moss carpets of boreal forests. Nature 419, 917–920. 10.1038/nature01051, PMID: 12410308

[ref16] Dunn O. J. (1965). A note on multiple comparisons using rank sums. Technometrics 7, 255–256. 10.1080/00401706.1965.10490253

[ref17] Engene N. Coates R. C. Gerwick W. H. (2010). 16S rRNA gene heterogeneity in the filamentous marine cyanobacterial genus *Lyngbya*. J. Phycol. 46, 591–601. 10.1111/j.1529-8817.2010.00840.x

[ref18] Engene N. Gerwick W. H. (2011). Intra-genomic 16S rRNA gene heterogeneity in cyanobacterial genomes. Fottea 11, 17–24. 10.5507/fot.2011.003

[ref19] Furuki T. Maeda S. Imajo S. Hiroi T. Amaya T. Hirokawa T. (2003). Rapid and selective extraction of phycocyanin from *Spirulina platensis* with ultrasonic cell disruption. J. Appl. Phycol. 15, 319–324. 10.1023/A:1025118516888

[ref84] Fogg G. E. (1998). The biology of polar habitats. New York, NY: Oxford University Press.

[ref20] Gentili F. Nilsson M. C. Zackrisson O. DeLuca T. H. Sellstedt A. (2005). Physiological and molecular diversity of feather moss associative N_2_-fixing cyanobacteria. J. Exp. Bot. 56, 3121–3127. 10.1093/jxb/eri309 16263908

[ref21] Gornall J. L. Jónsdóttir I. S. Woodin S. J. Van Der Wal R. (2007). Arctic mosses govern below-ground environment and ecosystem processes. Oecologia 153, 931–941. 10.1007/s00442-007-0785-0, PMID: 17618466

[ref85] Grubbs F. E. (1969). Procedures for detecting outlying observations in samples. Technometrics 11, 1–21. 10.1080/00401706.1969.10490657

[ref22] Gundale M. J. Deluca T. H. Nordin A. (2011). Bryophytes attenuate anthropogenic nitrogen inputs in boreal forests. Glob. Chang. Biol. 17, 2743–2753. 10.1111/j.1365-2486.2011.02407.x

[ref23] Gundale M. J. Wardle D. A. Nilsson M. C. (2012). The effect of altered macroclimate on N-fixation by boreal feather mosses. Biol. Lett. 8, 805–808. 10.1098/rsbl.2012.0429, PMID: 22696285 PMC3440997

[ref24] Hardy R. W. F. Holsten R. D. Jackson E. K. Burns R. C. (1968). The acetylene-ethylene assay for N_2_ fixation: laboratory and field evaluation. Plant Physiol. 43, 1185–1207. 10.1104/pp.43.8.1185, PMID: 16656902 PMC1086994

[ref25] Harper K. Boudreault C. DeGrandpré L. Drapeau P. Gauthier S. Bergeron Y. (2003). Structure, composition, and diversity of old-growth black spruce boreal forest of the clay belt region in Quebec and Ontario. Environ. Rev. 11, S79–S98. 10.1139/a03-013

[ref26] Herrera A. Boussiba S. Napoleone V. Hohlberg A. (1989). Recovery of C-phycocyanin from the cyanobacterium *Spirulina maxima*. J. Appl. Phycol. 1, 325–331. 10.1007/BF00003469

[ref27] Horváth H. Kovács A. W. Riddick C. Présing M. (2013). Extraction methods for phycocyanin determination in freshwater filamentous cyanobacteria and their application in a shallow lake. Eur. J. Phycol. 48, 278–286. 10.1080/09670262.2013.821525

[ref28] Houle D. Gauthier S. B. Paquet S. Planas D. Warren A. (2006). Identification of two genera of N_2_-fixing cyanobacteria growing on three feather moss species in boreal forests of Quebec. Can. J. Bot. 84, 1025–1029. 10.1139/b06-059

[ref29] Ininbergs K. Bay G. Rasmussen U. Wardle D. A. Nilsson M. C. (2011). Composition and diversity of *nifH* genes of nitrogen-fixing cyanobacteria associated with boreal forest feather mosses. New Phytol. 192, 507–517. 10.1111/j.1469-8137.2011.03809.x, PMID: 21714790

[ref30] Izydorczyk K. Tarczynska M. Jurczak T. Mrowczynski J. Zalewski M. (2005). Measurement of phycocyanin fluorescence as an online early warning system for cyanobacteria in reservoir intake water. Environ. Toxicol. 20, 425–430. 10.1002/tox.20128, PMID: 16007662

[ref31] Jean M. E. Cassar N. Setzer C. Bellenger J. P. (2012). Short-term N_2_ fixation kinetics in a moss-associated cyanobacteria. Environ. Sci. Technol. 46, 8667–8671. 10.1021/es3018539, PMID: 22849538

[ref32] Jean M. Mack M. C. Johnstone J. F. (2018). Spatial and temporal variation in moss-associated dinitrogen fixation in coniferous- and deciduous-dominated alaskan boreal forests. Plant Ecol. 219, 837–851. 10.1007/s11258-018-0838-y

[ref33] Jespersen L. Strømdahl L. D. Olsen K. Skibsted L. H. (2005). Heat and light stability of three natural blue colorants for use in confectionery and beverages. Eur. Food Res. Technol. 220, 261–266. 10.1007/s00217-004-1062-7

[ref86] Kirk J. T. O. (1994). Light and photosynthesis in aquatic ecosystems. Cambridge University Press.

[ref34] Kissoudi M. Sarakatsianos I. Samanidou V. (2018). Isolation and purification of food-grade C-phycocyanin from *Arthrospira platensis* and its determination in confectionery by HPLC with diode array detection. J. Sep. Sci. 41, 975–981. 10.1002/jssc.201701151, PMID: 29193817

[ref35] Kruskal W. H. Wallis W. A. (1952). Use of ranks in one-criterion variance analysis. J. Am. Stat. Assoc. 47, 583–621. 10.1080/01621459.1952.10483441

[ref36] Lawrenz E. Fedewa E. J. Richardson T. L. (2011). Extraction protocols for the quantification of phycobilins in aqueous phytoplankton extracts. J. Appl. Phycol. 23, 865–871. 10.1007/s10811-010-9600-0

[ref37] Lee N. K. Oh H. M. Kim H. S. Ahn C. Y. (2017). Higher production of C-phycocyanin by nitrogen-free (diazotrophic) cultivation of *Nostoc* sp. NK and simplified extraction by dark-cold shock. Bioresour. Technol. 227, 164–170. 10.1016/j.biortech.2016.12.053, PMID: 28024193

[ref38] Leppänen S. M. Salemaa M. Smolander A. Mäkipää R. Tiirola M. (2013). Nitrogen fixation and methanotrophy in forest mosses along a N deposition gradient. Environ. Exp. Bot. 90, 62–69. 10.1016/j.envexpbot.2012.12.006

[ref39] Li X. Dreher T. W. Li R. (2016). An overview of diversity, occurrence, genetics and toxin production of bloom-forming *Dolichospermum* (*Anabaena*) species. Harmful Algae 54, 54–68. 10.1016/j.hal.2015.10.015, PMID: 28073482

[ref40] Lindo Z. Whiteley J. A. (2011). Old trees contribute bio-available nitrogen through canopy bryophytes. Plant Soil 342, 141–148. 10.1007/s11104-010-0678-6

[ref41] Liu J. Liu W. Long X. E. Chen Y. Huang T. Huo J. (2020). Effects of nitrogen addition on C:N:P stoichiometry in moss crust-soil continuum in the N-limited gurbantünggüt desert, Northwest China. Eur. J. Soil Biol. 98:103174. 10.1016/j.ejsobi.2020.103174

[ref42] Lloyd A. H. Bunn A. G. (2007). Responses of the circumpolar boreal forest to 20th century climate variability. Environ. Res. Lett. 2:45013. 10.1088/1748-9326/2/4/045013

[ref43] Luthin J. N. Guymon G. L. (1974). Soil moisture-vegetation-temperature relationships in Central Alaska. J. Hydrol. 23, 233–246. 10.1016/0022-1694(74)90005-5

[ref44] MacColl R. (1998). Cyanobacterial phycobilisomes. J. Struct. Biol. 124, 311–334. 10.1006/jsbi.1998.4062, PMID: 10049814

[ref45] McQuaid N. Zamyadi A. Prévost M. Bird D. F. Dorner S. (2011). Use of in vivo phycocyanin fluorescence to monitor potential microcystin-producing cyanobacterial biovolume in a drinking water source. J. Environ. Monit. 13, 455–463. 10.1039/c0em00163e, PMID: 21157617

[ref46] Nübel U. Garcia-Pichel F. Muyzer G. (1997). PCR primers to amplify 16S rRNA genes from cyanobacteria. Appl. Environ. Microbiol. 63, 3327–3332. 10.1128/AEM.63.8.3327-3332.1997, PMID: 9251225 PMC168636

[ref47] Oechel W. C. Van Cleve K. (1986). “The role of bryophytes in nutrient cycling in the taiga” in Forest ecosystems in the Alaskan taiga. eds. Van Cleve K. Chapin F. S. Flanagan P. W. Viereck L. A. Dyrness C. T. (New York, NY: Springer), 121–137.

[ref87] Ouranos (2015). Vers l’adaptation: Synthèse des connaissances sur les changements climatiques au Québec. Édition 2015. Montréal, Québec: Ouranos.

[ref48] Papadaki S. Kyriakopoulou K. Tzovenis I. Krokida M. (2017). Environmental impact of phycocyanin recovery from *Spirulina platensis* cyanobacterium. Innov. Food Sci. Emerg. Technol. 44, 217–223. 10.1016/j.ifset.2017.02.014

[ref49] Piron R. Bustamante T. Barriga A. Lagos N. (2019). Phycobilisome isolation and C-phycocyanin purification from the cyanobacterium *Aphanizomenon gracile*. Photosynthetica 57, 491–499. 10.32615/ps.2019.064

[ref50] Poza-Carrión C. Fernández-Valiente E. Piñas F. F. Leganés F. (2001). Acclimation of photosynthetic pigments and photosynthesis of the cyanobacterium *Nostoc* sp. strain UAM206 to combined fluctuations of irradiance, pH, and inorganic carbon availability. J. Plant Physiol. 158, 1455–1461. 10.1078/0176-1617-00555

[ref51] Prates D. F. Radmann E. M. Duarte J. H. de Morais M. G. Costa J. A. V. (2018). *Spirulina* cultivated under different light emitting diodes: enhanced cell growth and phycocyanin production. Bioresour. Technol. 256, 38–43. 10.1016/j.biortech.2018.01.122, PMID: 29428612

[ref52] Price D. T. Alfaro R. I. Brown K. J. Flannigan M. D. Fleming R. A. Hogg E. H. (2013). Anticipating the consequences of climate change for Canada’s boreal forest ecosystems. Environ. Rev. 21, 322–365. 10.1139/er-2013-0042

[ref88] R Core Team (2017). R: A language and environment for statistical computing. R Foundation for Statistical Computing, Vienna, Austria. Available at: https://www.R-project.org/ (Accessed September 1, 2020).

[ref53] Raposo F. Barceló D. (2020). Challenges and strategies of matrix effects using chromatography-mass spectrometry an overview from research versus regulatory viewpoints. TrAC-Trends Anal. Chem. 10.1016/j.trac.2020.116068 (in press).

[ref54] Raposo F. Ibelli-Bianco C. (2020). Performance parameters for analytical method validation: controversies and discrepancies among numerous guidelines. TrAC-Trends Anal. Chem. 129:115913. 10.1016/j.trac.2020.115913

[ref55] Reis A. Mendes A. Lobo-Fernandes H. Empis J. A. Novais J. M. (1998). Production, extraction and purification of phycobiliproteins from *Nostoc* sp. Bioresour. Technol. 66, 181–187. 10.1016/S0960-8524(98)00064-9

[ref56] Reksten S. S. (2014). Sonication as a method for dislodging cyanobacteria from feather mosses. [Research project]. Reykjavík: University of Iceland.

[ref57] Rippka R. Deruelles J. Waterbury J. B. Herdman M. Stanier R. Y. (1979). Generic assignments, strain histories and properties of pure cultures of cyanobacteria. Microbiology 111, 1–61. 10.1099/00221287-111-1-1

[ref58] Rousk K. Degboe J. Michelsen A. Bradley R. Bellenger J. P. (2017). Molybdenum and phosphorus limitation of moss-associated nitrogen fixation in boreal ecosystems. New Phytol. 214, 97–107. 10.1111/nph.14331 27883187

[ref59] Rousk K. DeLuca T. H. Rousk J. (2013). The cyanobacterial role in the resistance of feather mosses to decomposition-toward a new hypothesis. PLoS One 8:e62058. 10.1371/journal.pone.0062058, PMID: 23614013 PMC3626682

[ref60] Rousk K. Michelsen A. (2017). Ecosystem nitrogen fixation throughout the snow-free period in subarctic tundra: effects of willow and birch litter addition and warming. Glob. Chang. Biol. 23, 1552–1563. 10.1111/gcb.13418, PMID: 27391280

[ref61] Royston P. (1995). Remark AS R94: a remark on algorithm AS 181: the W-test for normality. Appl. Stat. 44, 547–551. 10.2307/2986146

[ref62] Santiago-Santos M. C. Ponce-Noyola T. Olvera-Ramírez R. Ortega-López J. Cañizares-Villanueva R. O. (2004). Extraction and purification of phycocyanin from *Calothrix* sp. Process Biochem. 39, 2047–2052. 10.1016/j.procbio.2003.10.007

[ref63] Sarada R. Pillai M. G. Ravishankar G. A. (1999). Phycocyanin from *Spirulina* sp.: influence of processing of biomass on phycocyanin yield, analysis of efficacy of extraction methods and stability studies on phycocyanin. Process Biochem. 34, 795–801. 10.1016/S0032-9592(98)00153-8

[ref64] Schalles J. F. Yacobi Y. Z. (2000). Remote detection and seasonal patterns of phycocyanin, carotenoid and chlorophyll pigments in eutrophic waters. Ergebnisse der Limnol. 55, 153–168.

[ref65] Schlüter L. Garde K. Kaas H. (2004). Detection of the toxic cyanobacteria *Nodularia spumigena* by means of a 4-keto-myxoxanthophyll-like pigment in the Baltic Sea. Mar. Ecol. Prog. Ser. 275, 69–78. 10.3354/meps275069

[ref66] Scott D. L. Bradley R. L. Bellenger J. P. Houle D. Gundale M. J. Rousk K. (2018). Anthropogenic deposition of heavy metals and phosphorus may reduce biological N_2_ fixation in boreal forest mosses. Sci. Total Environ. 630, 203–210. 10.1016/j.scitotenv.2018.02.192 29477819

[ref67] Seppälä J. Ylöstalo P. Kaitala S. Hällfors S. Raateoja M. Maunula P. (2007). Ship-of-opportunity based phycocyanin fluorescence monitoring of the filamentous cyanobacteria bloom dynamics in the Baltic Sea. Estuar. Coast. Shelf Sci. 73, 489–500. 10.1016/j.ecss.2007.02.015

[ref68] Sigurdsson B. D. Medhurst J. L. Wallin G. Eggertsson O. Linder S. (2013). Growth of mature boreal Norway spruce was not affected by elevated [CO_2_] and/or air temperature unless nutrient availability was improved. Tree Physiol. 33, 1192–1205. 10.1093/treephys/tpt043, PMID: 23878169

[ref69] Sloth J. K. Wiebe M. G. Eriksen N. T. (2006). Accumulation of phycocyanin in heterotrophic and mixotrophic cultures of the acidophilic red alga *Galdieria sulphuraria*. Enzyme Microb. Technol. 38, 168–175. 10.1016/j.enzmictec.2005.05.010

[ref70] Stanier R. Y. Cohen-Bazire G. (1977). Phototrophic prokaryotes: the cyanobacteria. Annu. Rev. Microbiol. 31, 225–274. 10.1146/annurev.mi.31.100177.001301, PMID: 410354

[ref71] Stewart D. E. Farmer F. H. (1984). Extraction, identification, and quantitation of phycobiliprotein pigments from phototrophic plankton. Limnol. Oceanogr. 29, 392–397. 10.4319/lo.1984.29.2.0392

[ref72] Tagesson T. Schurgers G. Horion S. Ciais P. Tian F. Brandt M.. (2020). Recent divergence in the contributions of tropical and boreal forests to the terrestrial carbon sink. Nat. Ecol. Evol. 4, 202–209. 10.1038/s41559-019-1090-0, PMID: 31988446

[ref73] Tavanandi H. A. Mittal R. Chandrasekhar J. Raghavarao K. S. M. S. (2018). Simple and efficient method for extraction of C-phycocyanin from dry biomass of *Arthospira platensis*. Algal Res. 31, 239–251. 10.1016/j.algal.2018.02.008

[ref74] Thompson M. Wood R. (1993). International harmonized protocol for proficiency testing of (chemical) analytical laboratories. J. AOAC Int. 76, 926–940. 10.1093/jaoac/76.4.926

[ref75] Turetsky M. R. (2003). The role of bryophytes in carbon and nitrogen cycling. Bryologist 106, 395–409. 10.1639/05

[ref76] Turetsky M. R. Bond-Lamberty B. Euskirchen E. Talbot J. Frolking S. McGuire A. D.. (2012). The resilience and functional role of moss in boreal and arctic ecosystems. New Phytol. 196, 49–67. 10.1111/j.1469-8137.2012.04254.x, PMID: 22924403

[ref77] Turetsky M. R. Mack M. C. Hollingsworth T. N. Harden J. W. (2010). The role of mosses in ecosystem succession and function in Alaska’s boreal forest. Can. J. For. Res. 40, 1237–1264. 10.1139/X10-072

[ref78] Ürek R. O. Tarhan L. (2012). The relationship between the antioxidant system and phycocyanin production in *Spirulina maxima* with respect to nitrate concentration. Turk. J. Bot. 36, 369–377. 10.3906/bot-1106-1

[ref79] Wardle D. A. Jonsson M. Bansal S. Bardgett R. D. Gundale M. J. Metcalfe D. B. (2011). Linking vegetation change, carbon sequestration and biodiversity: insights from island ecosystems in a long-term natural experiment. J. Ecol. 100, 16–30. 10.1111/j.1365-2745.2011.01907.x

[ref80] Warshan D. Bay G. Nahar N. Wardle D. A. Nilsson M. C. Rasmussen U. (2016). Seasonal variation in *nifH* abundance and expression of cyanobacterial communities associated with boreal feather mosses. ISME J. 10, 2198–2208. 10.1038/ismej.2016.17, PMID: 26918665 PMC4989308

[ref81] Whiteley J. A. Gonzalez A. (2016). Biotic nitrogen fixation in the bryosphere is inhibited more by drought than warming. Oecologia 181, 1243–1258. 10.1007/s00442-016-3601-x, PMID: 27098528

[ref82] Wilson J. A. Coxson D. S. (1999). Carbon flux in a subalpine spruce-fir forest: pulse release from *Hylocomium splendens* feather-moss mats. Can. J. Bot. 77, 564–569. 10.1139/b99-002

[ref83] Zackrisson O. Deluca T. H. Gentili F. Sellstedt A. Jäderlund A. (2009). Nitrogen fixation in mixed *Hylocomium splendens* moss communities. Oecologia 160, 309–319. 10.1007/s00442-009-1299-8, PMID: 19252932

