# Supplementary material for: Quantification of Moss-Associated Cyanobacteria Using Phycocyanin Pigment Extraction
Source: Front Microbiol. 2021 Jan 5;11:611792. doi: 10.3389/fmicb.2020.611792 (PMC7813775; doi:10.3389/fmicb.2020.611792)
Supplement: Supplementary file 1 [file Data_Sheet_1.pdf]

Quantification of moss-associated cyanobacteria using phycocyanin pigment extraction

Marie Renaudin[a], Romain Darnajoux[b], Jean-Philippe Bellenger[a*]

[a] Centre Sève, Département de Chimie, Université de Sherbrooke, J1K 2R1, Qc, Canada

[b] Department of Geosciences, Princeton University, 08544, NJ, USA

**: jean-philippe.bellenger@usherbrooke.ca*

Supplementary material

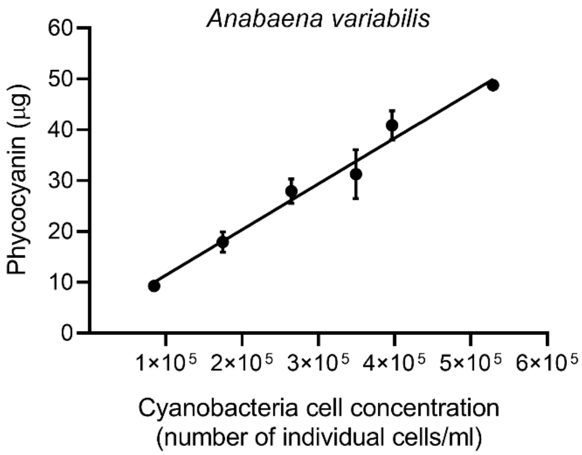

Figure S1. Phycocyanin mass mean ± SD (n=3) linearity in *Anabaeana variabilis* culture. The straight line represents the linear regression between phycocyanin mass and the number of individual cyanobacteria cell per ml of culture.

Table S1. Phycocyanin concentrations (µg.g^{-1} moss DW ± SD) measured in *Ptilium crista-castrensis* and *Pleurozium schreberi* collected in June and September 2019 in Quebec, Canada (n = 3).

		**Sampling site**			
		**1**	**2**	**3**	**4**
**June**	*Ptilium crista-castrensis*	0.85 ± 0.25	1.26 ± 0.42	0.79 ± 0.02	0.45 ± 0.05
	Pleurozium schreberi	0.48 ± 0.10	0.47 ± 0.08	0.46 ± 0.03	0.41 ± 0.02
**September**	*Ptilium crista-castrensis*	0.71 ± 0.23	0.96 ± 0.17	0.75 ± 0.08	0.56 ± 0.10
	Pleurozium schreberi	0.88 ± 0.43	0.72 ± 0.04	0.71 ± 0.17	0.61 ± 0.11
